# *In ovo* Administration of Defined Lactic Acid Bacteria Previously Isolated From Adult Hens Induced Variations in the Cecae Microbiota Structure and *Enterobacteriaceae* Colonization on a Virulent *Escherichia coli* Horizontal Infection Model in Broiler Chickens

**DOI:** 10.3389/fvets.2020.00489

**Published:** 2020-08-21

**Authors:** Margarita A. Arreguin-Nava, Brittany D. Graham, Bishnu Adhikari, Melissa Agnello, Callie M. Selby, Xochitl Hernandez-Velasco, Christine N. Vuong, Bruno Solis-Cruz, Daniel Hernandez-Patlan, Juan D. Latorre, Guillermo Tellez, Billy M. Hargis, Guillermo Tellez-Isaias

**Affiliations:** ^1^Eco-Bio LLC, Fayetteville, AR, United States; ^2^Department of Poultry Science, University of Arkansas, Fayetteville, AR, United States; ^3^uBiome, Inc., San Francisco, CA, United States; ^4^Departamento de Medicina y Zootecnia de Aves, Facultad de Medicina Veterinaria y Zootecnia, Universidad Nacional Autonoma de Mexico (UNAM), Mexico City, Mexico; ^5^Laboratorio 5: LEDEFAR, Unidad de Investigación Multidisciplinaria, FES Cuautitlán, UNAM, Cuautitlán Izcalli, Mexico

**Keywords:** broiler, *Escherichia coli*, hatcher, *in ovo*, probiotic

## Abstract

The effects of *in ovo* administration of a defined lactic acid microbiota (LAM), previously isolated from adult hens, in the cecae microbiota structure and *Enterobacteriaceae* colonization after exposure to virulent *Escherichia coli* during the hatching phase of broiler chickens were evaluated. Embryos inoculated with LAM showed a significant (*P* < 0.05) reduction of *Enterobacteriaceae* colonization at day-of-hatch (DOH) and day (d) 7. Furthermore, there was a significant increase in total lactic acid bacteria on DOH, body weight (BW) DOH, BW d7, and d0–d7 BW gain and reduced mortality d0–d7 was observed in the LAM group compared with that in phosphate-buffered saline (PBS) control. The bacterial composition at the family level revealed that the *Enterobacteriaceae* was numerically reduced, whereas the *Ruminococcaceae* was significantly increased in the LAM group when compared with that in the PBS control. Moreover, the bacterial genera *Proteus* and *Butyricicoccus* and unidentified bacterial genera of family *Lachnospiraceae* and *Erysipelotrichaceae* were significantly enriched in the LAM group. In contrast, the *Clostridium* of the family *Peptostreptococcaceae* and unidentified genus of family *Enterobacteriaceae* were significantly abundant in the PBS control group. In summary, *in ovo* administration of a defined LAM isolated from adult hens did not affect hatchability, improved body weight gain and reduced mortality at d7, induced variations in the cecae microbiota structure and reduced *Enterobacteriaceae* colonization on a virulent *E. coli* horizontal infection model in broiler chickens.

## Introduction

Early establishment of the gastrointestinal microbiota has been shown to have significant benefits in the development of gut-associated lymphoid tissues and intestinal integrity (1, 2). Therefore, several investigators have decided to evaluate the administration of *in ovo* probiotics in chickens. Several studies have indicated that this method does not alter hatchability, improves intestinal health, and favors microbial diversity (3–5). Furthermore, *in ovo* delivery of probiotics may have a significant impact in commercial poultry because hatching cabinets represent one of the first potential sources of pathogenic enterobacteria (6, 7). Avian pathogenic *Escherichia coli* (APEC) may also penetrate the shell (8) or can be vertically transferred (9), causing significant mortality during the first week (10). Recently, our laboratory has developed a novel *in ovo* challenge model for APEC strains (11). The objective of the present study was to evaluate the *in ovo* administration of defined lactic acid microbiota (LAM), previously isolated from adult hens, on hatchability, performance during the first 7 days after hatch, microbiota composition, and *Enterobacteriaceae* colonization while utilizing a virulent *E. coli* horizontal infection model in broiler chickens.

## Materials and Methods

### Isolation and Selection of Lactic Acid Microbiota Isolated From Adult Hens

Ten lactic acid bacteria (LAB) were isolated from 10 34-week-old Hy-Line Brown backyard flock hens fed with a maize grain diet. Cecal and ileum (Meckel diverticulum to cecal tonsils) content from these birds was collected, and then, both sections were flushed with phosphate-buffered saline (PBS). Epithelium and intestinal contents were briefly homogenized, serially diluted, and plated on de Man–Rogosa–Sharpe (MRS) agar plates (Catalog no. 288110, Becton Dickinson and Co., Sparks, MD 21152 USA) to obtain one pure colony from each sample. The isolates were identified by 16S ribosomal RNA (rRNA) sequence analyses (Microbial ID Inc., Newark, DE 19713, USA). The report showed that four of the strains were *Lactobacillus johnsonii*, three isolates were *Weissella confusa*, two *Lactobacillus salivarius*, and one as *Pediococcus parvulus*. Aliquots of the combined culture containing 10 selected LAB isolates were grown on MRS agar as a combined batch culture (LAM) and used in the present study.

### *Escherichia coli* Culture and Challenge

Dr. A. M. Donoghue, from the Poultry Production and Product Safety Research Unit, United States Department of Agriculture—Agricultural Research Service, kindly donated the APEC strain that was used in these experiments (12). This *E. coli* isolate, obtained from adult chickens with colibacillosis, was confirmed to be susceptible to tetracycline and oxytetracycline (Animal Disease Diagnostic Laboratory, Ohio Department of Agriculture, Reynoldsburg, USA). This strain was serially diluted to the desired colony-forming unit (CFU) concentration for *in ovo* challenge (day [d] 19 of embryogenesis) as described previously (11).

### Enumeration of Bacteria

In trials 1 and 2, the gastrointestinal tract (GIT) (duodenum to the cecum) was aseptically removed postmortem and collected into sterile bags. These samples were then diluted and plated on either MRS agar (Difco Lactobacilli MRS Agar, cat. no. 90004-084, VWR, Suwanee, USA) to evaluate the total number of LAB or MacConkey agar (VWR cat. no. 89429-342 Suwanee, USA) to evaluate the number of gram-negative bacteria as described by Tellez et al. (13). To confirm negative results or account for the possibility that bacterial groups were present in lower numbers, the detection limit on bacterial recovery by direct plating was confirmed by enrichment of the samples on selective media, respectively.

### Experimental Design

In the present study, two independent trials were conducted following the previously published *in ovo* challenge model for virulent *E. coli* (11). In each trial, 360 18-day-old Ross 308 embryos were candled, randomly allocated, and placed into two separate hatchers (GQF 1550 Digital Cabinet Egg Incubator) based on treatment group (*n* = 180/treatment group). In both trials, the same hatchers, set in the same room, were used for each experimental treatment. On d19 of embryogenesis, embryos were inoculated, into the amnion, with either 0.2 ml with sterile PBS control or 10^7^ CFU/ml of LAM as described previously (3). Additionally, on d19 of embryogenesis, seeder embryos (*n* = 18 seeders/hatcher or 10%/hatcher) were inoculated with *E. coli*/tetracycline treatment (4.5 × 10^4^ CFU/ml *E. coli* + 272 μg/ml) via *in ovo* injection into the amnion and segregated into mesh hatching bags (reusable mesh nylon netting, IDS, Amazon). Doses for coadministration of tetracycline and this particular virulent *E. coli* strain have been described previously (11). On d21, dry chicks were removed from hatchers, and hatchability was recorded. For each trial, GIT samples were collected postmortem on day-of-hatch (DOH) and d7 to evaluate gastrointestinal composition on selective media for enumeration of total presumptive gram-negative or total aerobic LAB as previously published (14). From each trial, 90 chicks from each group were neck-tagged, individually weighed, and randomly allocated into three-floor pens (*n* = 30 chicks/pen) and provided *ad libitum* access to water and a balanced, unmedicated corn and soybean diet (Table 1). Weight allocation on DOH was performed to normalize body weight (BW) and prevent initial treatment effect on BW as previously described (11). Mortality was recorded for the duration of each trial (7-day trial period) as well as BW gain (BWG). Cecal contents were collected from six chickens per group to evaluate microbiome analysis (trial 2 only). Chickens were provided *ad libitum* access to water and a balanced, unmedicated corn and soybean diet, meeting the nutritional requirements for broilers recommended by Aviagen. This study was carried out following the recommendations of the Institutional Animal Care and Use Committee at the University of Arkansas, Fayetteville. The Institutional Animal Care and Use Committee approved protocol #17073 at the University of Arkansas, Fayetteville, for this study.

**Table 1:** Ingredient composition and nutrient content of a corn–soybean starter diet used in all experimental groups on an as-is basis.

**Item**	**Starter diet**
**Ingredients (%)**	
Corn	57.34
Soybean meal	34.66
Poultry fat	3.45
Dicalcium phosphate	1.86
Calcium carbonate^a^	0.99
Salt	0.38
DL-Methionine	0.33
L-Lysine HCl	0.31
Threonine	0.16
Vitamin premix^b^	0.20
Mineral premix^c^	0.10
Choline chloride 60%	0.20
Antioxidant^d^	0.02
**Calculated analysis**	
Metabolizable energy (kcal/ kg)	3,035
Crude protein (%)	22.16
Ether extract (%)	5.68
Lysine (%)	1.35
Methionine (%)	0.64
Methionine + cystine (%)	0.99
Threonine (%)	0.92
Tryptophan (%)	0.28
Total calcium	0.90
Available phosphorus	0.45
**Determined analysis**	
Crude protein (%)	21.15
Ether extract (%)	6.05
Calcium (%)	0.94
Phosphorus (%)	0.73

a *Inclusion of 10^6^ spores/g of feed mixed with calcium carbonate*.

b *Vitamin premix supplied the following per kilogram: vitamin A, 20,000 IU; vitamin D3, 6,000 IU; vitamin E, 75 IU; vitamin K3, 6.0 mg; thiamine, 3.0 mg; riboflavin, 8.0 mg; pantothenic acid, 18 mg; niacin, 60 mg; pyridoxine, 5 mg; folic acid, 2 mg; biotin, 0.2 mg; cyanocobalamin, 16 μg; and ascorbic acid, 200 mg (Nutra Blend LLC, Neosho, MO 64850)*.

c *Mineral premix supplied the following per kilogram: manganese, 120 mg; zinc, 100 mg; iron, 120 mg; copper, 10 to 15 mg; iodine, 0.7 mg; selenium, 0.4 mg; and cobalt, 0.2 mg (Nutra Blend LLC, Neosho, MO 64850)*.

d *Ethoxyquin*.

### Microbiota Analysis

#### Sample Processing, DNA Extraction, PCR, Library Preparation, and Sequencing

At d7, ceca content samples (*n* = 6/group) were prepared and transferred into collection tubes containing a lysis and stabilization buffer. DNA extraction, amplification, and library preparation were performed as described by Almonacid et al. (15). Briefly, samples were lysed through bead-beating, and DNA was extracted by guanidine thiocyanate silica column-based purification method using a liquid-handling robot in a class 1,000 cleanroom (16). The following universal primers were used for PCR amplification of the V4 variable region of the 16S rRNA gene: (515F: 5′GTGCCAGCMGCCGCGGTAA and 806R: 5′GGACTACHVGGGTWTCTAAT); primers also contained Illumina tags, and barcodes were used for amplification of the V4 variable region of the 16S rRNA gene (17). PCR products were then pooled, column-purified, and size-selected through microfluidic DNA fractionation (18). Consolidated libraries were quantified by quantitative real-time PCR using the Kapa Bio-Rad iCycler qPCR kit on a BioRad MyiQ before loading for sequencing. Sequencing was performed in a pair-end modality on the Illumina NextSeq 500 platform rendering 2 × 150-bp pair-end sequences.

#### 16S Ribosomal RNA Gene Sequences Analysis

After sequencing, the samples were demultiplexed, utilizing Illumina's BCL2FASTQ algorithm. Forward and reverse reads obtained in each of the four lanes per sample were filtered using the following criteria: both forward and reverse reads in a pair must have an average Q-score > 30. Primers and any leading random nucleotides (used to increase the diversity of the library being sequenced) were trimmed, forward reads were capped at 125 bp, and reverse reads were capped at 124 bp. After quality filtering as described earlier, the Deblur (19) workflow was applied for the forward reads to generate a feature table and representative sequences using the “qiime deblur denoise-16S” method implemented in QIIME2 version 2019.1 (20). The features that were present only in a single sample were removed from the feature table. Naive Bayes classifier (21) was trained using Green genes 13_8 99% operational taxonomic units (OTUs) (22), where the sequences were trimmed to include only 125 bases from the region of the 16S rRNA gene bound by the 515F/806R primer pair. This pretrained classifier was used to assign taxonomy to the representative sequences using the q2-feature-classifier plugin. Microbial diversity analyses were performed using the q2-diversity plugin of QIIME2 using the even sampling depth of 14,610. The alpha diversity as computed by observed OTU metric and Shannon's index (23) and beta diversity as calculated by unweighted UniFrac (24) distance metrics are reported. All figures were created using ggplot2 packages (25) on R version 3.5.3.

### Statistical Analysis

All data were subjected to one-way analysis of variance as a completely randomized design using the general linear model procedure of SAS (26). Data are expressed as mean ± standard error (SE). Significant differences (*P* < 0.05) among the means were further separated using Duncan's multiple range test for bacteria recovery, BW, and BWG. Hatchability and mortality were compared using the chi-square test of independence to determine the significance (*P* < 0.05) for these studies (27). The linear discriminant analysis effect size (LEfSe) method was used to identify significantly different bacterial taxa between two treatments at different levels of the taxonomy (phylum, family, and genus) using the criteria: *P* < 0.05, and LDA score (log10) > 2.0. For statistical analysis of alpha and beta diversity, Wilcoxon and permutational multivariate analysis of variance (PERMANOVA) (28) tests were used, respectively. In both tests, the level of significance was set at *P* < 0.05.

## Results

The effect of *in ovo* administration of a LAM on presumptive gram-negative and LAB recovered from the GIT in a virulent *E. coli* seeder is summarized in Table 2. In both trials, LAM significantly reduced (*P* < 0.05) the recovery of gram-negative bacteria from the GIT at hatch and at d7 when compared with the *in ovo* PBS control group. However, *in ovo* administration of LAM significantly increased the recovery of presumptive LAB on DOH when compared with control embryos in both trials (Table 2). No significant differences in the recovery of presumptive LAB were observed at d7 between control or treated groups (data not shown).

**Table 2:** Effect of *in ovo* administration of a lactic acid microbiota (LAM) on gram-negative and presumptive lactic acid bacteria recovered from the gastrointestinal tract (duodenum to the cecum) in an *in ovo* challenge model for horizontal transmission of a virulent *Escherichia coli*^1^.

**Treatment**	**Gram-negative recovery, day-of-hatch (Log_**10**_ CFU/g)**	**Presumptive lactic acid bacteria, day-of-hatch (Log_**10**_ CFU/g)**	**Gram-negative recovery, day 7 (Log_**10**_ CFU/g)**
**Trial 1**			
*In ovo* PBS control	4.32 ± 0.91^a^	5.17 ± 1.01^b^	7.43 ± 0.12^a^
*In ovo* 10^7^ CFU/ml LAM	2.19 ± 0.77^b^	8.44 ± 0.12^a^	4.21 ± 0.77^b^
**Trial 2**			
*In ovo* PBS control	3.91 ± 0.81^a^	1.84 ± 0.84^b^	6.34 ± 0.33^a^
*In ovo* 10^7^ CFU/ml LAM	2.12 ± 0.55^b^	8.60 ± 0.12^a^	3.10 ± 0.77^b^

1 *Data expressed as mean ± SE*.

a,b *Indicates significant difference between treatment groups within columns (P < 0.05)*.

*PBS, phosphate-buffered saline*.

Table 3 shows the results of the effect of *in ovo* administration of a LAM on hatchability, BW at DOH and d7, BWG, and d7 mortality. In both trials, no significant differences in hatchability were observed. Nevertheless, *in ovo* administration of the LAM significantly increased the average BW at hatch and at day 7, as well as BWG from d0 to d7 when compared with the control PBS group. Furthermore, a significant reduction in mortality was also observed in both trials in embryos that received the LAM when compared with the control PBS group (Table 3).

**Table 3:** Effect of *in ovo* administration of a lactic acid microbiota (LAM) on hatchability, body weights, and mortality at day 7, an *in ovo* challenge model for horizontal transmission of a virulent *Escherichia coli*.

**Group**	**Hatchability^1^**	**Average BW day-of-hatch**	**Average BW Day 7**	**BW gain Day 0–7**	**Mortality^2^ Day 7**
**Trial 1**					
*In ovo* PBS control	174/180 (96.66%)	40.03 ± 0.07^b^	164.56 ± 2.52^b^	116.93 ± 2.63^b^	14/90 (15.55%)^*^
*In ovo* 10^7^ CFU/ml LAM	177/180 (98.33%)	47.87± 0.65^a^	185.14 ± 2.71^a^	137.27 ± 2.69^a^	5/90 (5.55%)
**Trial 2**					
*In ovo* PBS control	176/180 (97.77%)	41.30 ± 0.03^b^	161.31 ± 2.68^b^	111.81 ± 1.91^b^	16/90 (17.77%)^*^
*In ovo* 10^7^ CFU/ml LAM	179/180 (99.44%)	45.77± 0.11^a^	175.15 ± 2.71^a^	129.38 ± 3.12^a^	7/90 (7.77%)

1 *Data expressed as number of chicks that hatched/total number of 18-day embryos placed (%), n = 180 embryos*.

2 *Data expressed as number of chicks that died from placement to 7 days/total number placed (%), n = 90 (3 replicates, n = 30/replicate)*.

a,b *Indicates significant difference (P < 0.05). Data expressed as mean ± SE*.

* *Indicates significant differences in mortality (P < 0.05)*.

*PBS, phosphate-buffered saline*.

### Summary of the Feature Table

The summarization of the feature table resulted in 703,667 sequence reads in 11 samples (5 PBS control and 6 LAM) that range from 14,610 to 99,834 reads per sample. The median and mean ± SE reads per sample were 66,975 and 63,969.73 ± 7,716.50, respectively. Moreover, there were altogether 102 unique features (amplicon sequence variants) from all samples.

### Bacterial Composition at the Phylum Level

**Figure 1:**
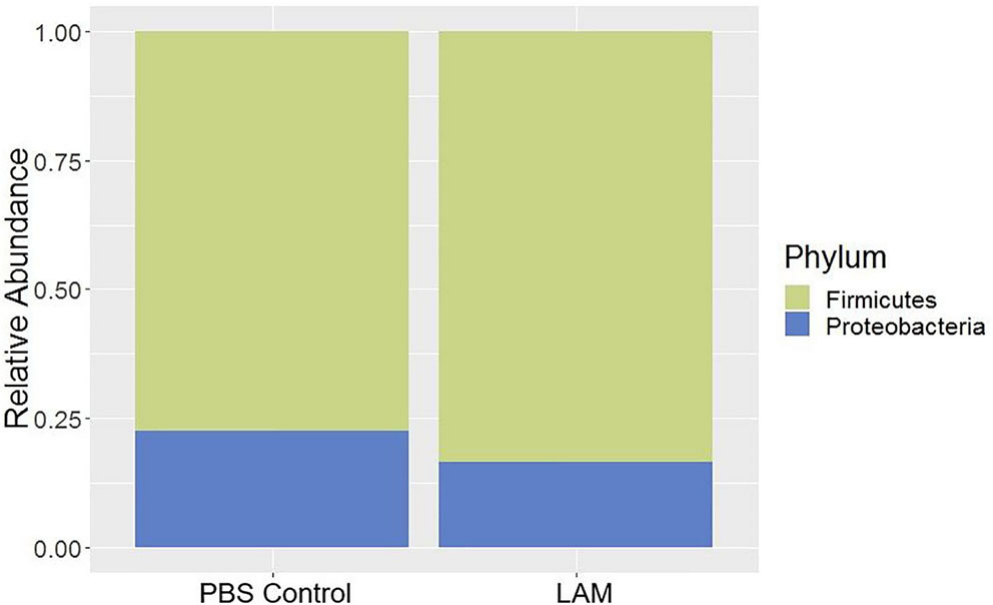
Bacterial phyla composition of phosphate-buffered saline control and LAM treatment groups in trial 2.

Firmicutes and Proteobacteria are the only two phyla detected, as shown in Figure 1. Firmicutes was reported as a dominant phylum in both groups (PBS control: 77.40 ± 3.03%, LAM: 83.56 ± 7.40%), followed by the Proteobacteria (PBS control: 22.59 ± 3.03%, LAM: 16.43 ± 7.40%). Although not significant, the Firmicutes were higher in LAM, whereas the Proteobacteria was higher in the PBS control group (Figure 1).

### Bacterial Composition at the Family Level

**Figure 2:**
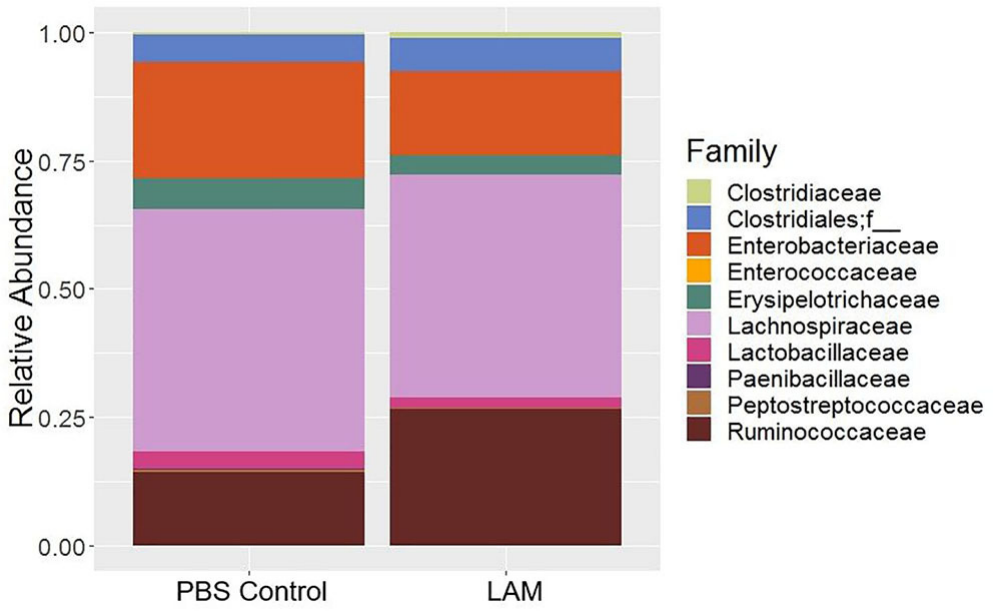
Bacterial family composition of phosphate-buffered saline control and LAM treatment groups in trial 2.

**Figure 3:**
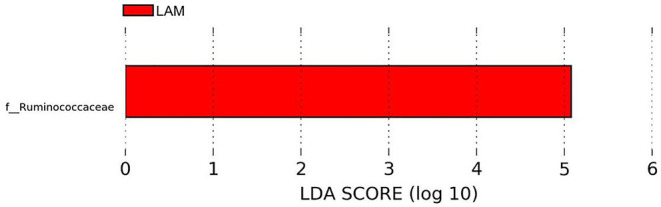
Differentially abundant bacterial families identified by LEfSe (*P* < 0.05 and LDA score >2.0). *Ruminococcaceae* was significantly enriched in the LAM treatment group as compared with that in the phosphate-buffered saline control in trial 2.

The relative abundance of bacterial families recovered from two treatment groups is shown in Figure 2. The relative abundance of *Lachnospiraceae* was found to be the highest in both groups (PBS control: 47.34 ± 5.42%, LAM: 43.46 ± 6.27%), followed by the *Enterobacteriaceae* in the PBS control group (22.59 ± 3.03%) and the *Ruminococcaceae* family in the LAM group (26.55 ± 6.89%). The relative abundance of the *Enterobacteriaceae* in the LAM and the relative abundance of *Ruminococcaceae* in the PBS control group were 16.43 ± 7.40% and 14.24 ± 5.53%, respectively. The other critical bacterial families were *Erysipelotrichaceae* (PBS control: 5.95 ± 3.11%, LAM: 3.73 ± 3.22%), an unidentified family of order Clostridiales (PBS control: 5.42 ± 3.46%, LAM: 6.46 ± 2.19%), Lactobacillaceae (PBS control: 3.20 ± 1.23, LAM: 2.05 ± 1.23), and Clostridiaceae (PBS control: 0.45 ± 0.35%, LAM: 1.05 ± 0.85%). Also, *Peptostreptococcaceae, Enterococcaceae*, and *Paenibacillaceae* were reported with an average relative abundance across all samples <1%. Interestingly, LEfSe analysis identified the bacterial family *Ruminococcaceae* to be significantly higher in the LAM group as compared with that in the PBS control group, as shown in Figure 3 (LEfSe, *P* < 0.05, LDA score > 2.0).

### Bacterial Composition at the Genus Level

**Figure 4:**
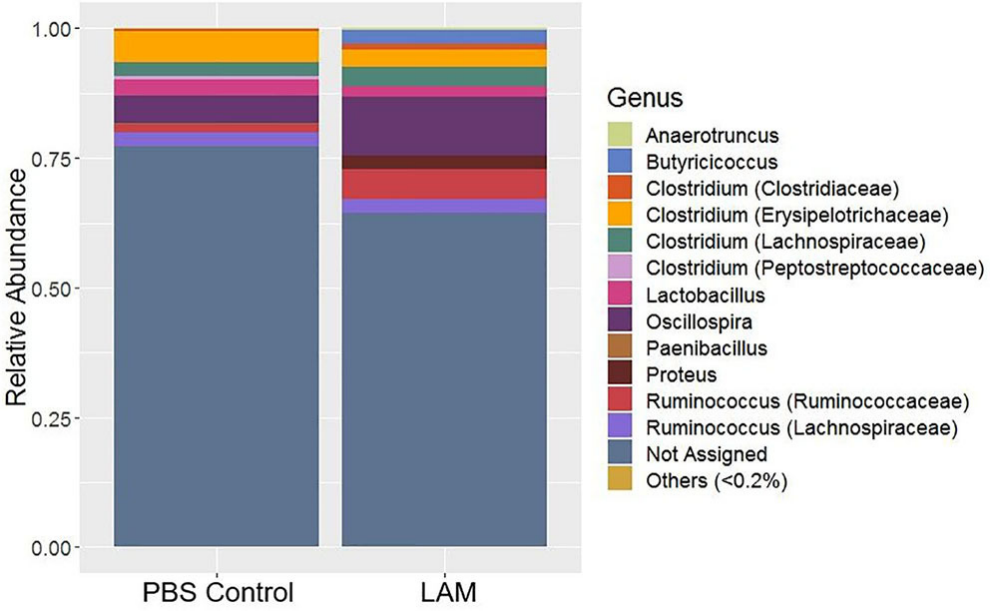
Composition of the bacterial genera that were found in phosphate-buffered saline control and LAM treatment groups. “Not Assigned” represents the sequence reads that were not assigned at any genus, however, were assigned at the higher level. Others represent the minor bacterial taxa whose average relative abundance across samples was <0.2% in trial 2.

The relative abundance of major bacterial genera identified in two treatment groups is shown in Figure 4. The majority of sequence reads (>50%) were not properly assigned at the genus level, as shown by the “Not Assigned” group (Figure 4) but were assigned at the higher level of the taxonomy. The relative abundance of minor bacterial genera (an average across all samples <0.2%) were grouped into “Others.” *Clostridium* genus belonging to the families Clostridiaceae (PBS control: 0.44 ± 0.35%, LAM: 1.02 ± 0.86%), *Erysipelotrichaceae* (PBS control: 5.89 ± 3.11%, LAM: 3.33 ± 3.19%), *Lachnospiraceae* (PBS control: 2.80 ± 0.44%, LAM: 3.84 ± 0.55%), and *Peptostreptococcaceae* (PBS control: 0.48 ± 0.25%, LAM not detected) were identified (Figure 4). Likewise, the genus *Ruminococcus* of the family *Lachnospiraceae* (PBS control: 2.73 ± 1.07%, LAM: 2.77 ± 1.21%) and *Ruminococcaceae* (PBS control: 1.48 ± 0.67%, LAM: 5.74 ± 2.42%) were found. Also, the genus *Oscillospira* that belongs to the family *Ruminococcaceae* has reported the highest percentage in the LAM group (11.25 ± 2.45%), whereas the second highest in the PBS control group (5.33 ± 1.53%). Another important observation was that the genera *Butyricicoccus* and *Proteus* were not detected in the PBS control group, whereas they were found in the LAM group 2.59 ± 1.37% and 2.51 ± 1.06%, respectively. The genera *Paenibacillus* and *Anaerotruncus* were reported <1% in each treatment group.

**Figure 5:**
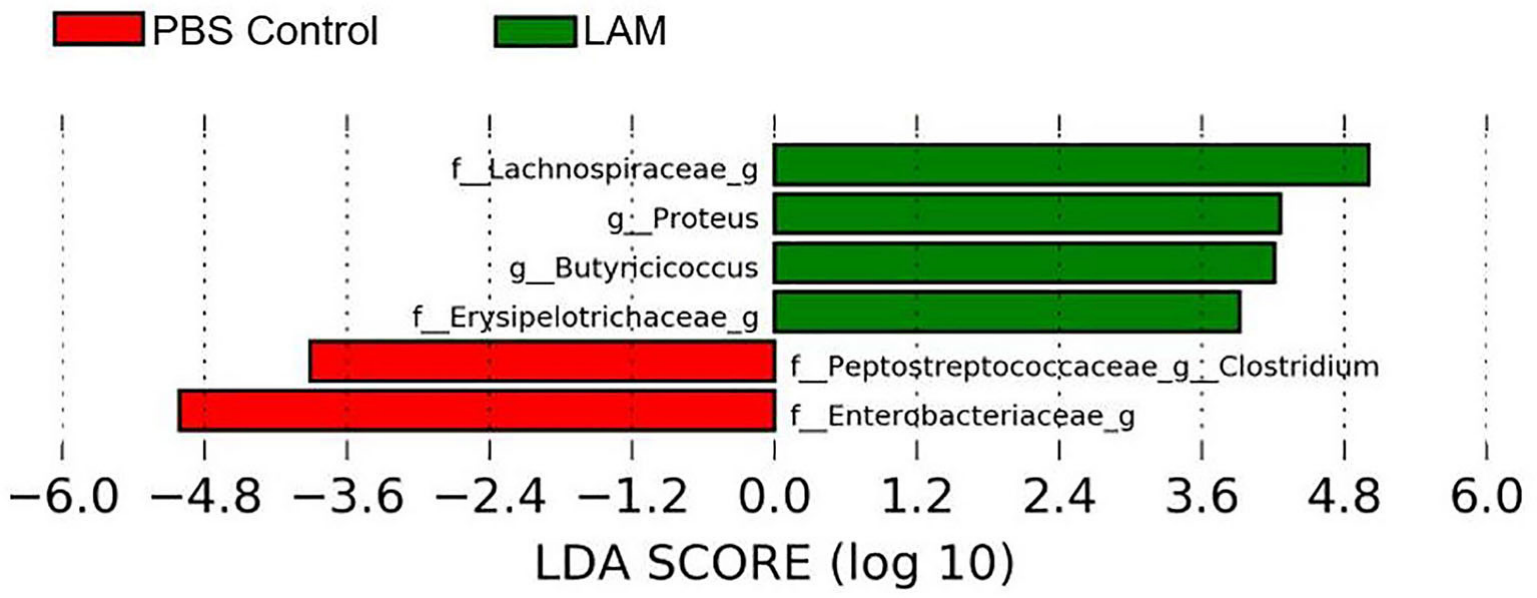
Differentially abundant bacterial genera identified by LEfSe (*P* < 0.05 and LDA score >2.0) between two treatment groups: phosphate-buffered saline control and LAM in trial 2.

The bacterial genera *Proteus, Butyricicoccus*, and unidentified bacterial genera of family *Lachnospiraceae* and *Erysipelotrichaceae* were significantly enriched in the LAM group. In contrast, the *Clostridium* of the family *Peptostreptococcaceae* and unidentified genus of family *Enterobacteriaceae* were significantly abundant in the PBS control group (LEfSe, *P* < 0.05 and LDA score > 2.0) (Figure 5).

### Alpha Diversity

**Figure 6:**
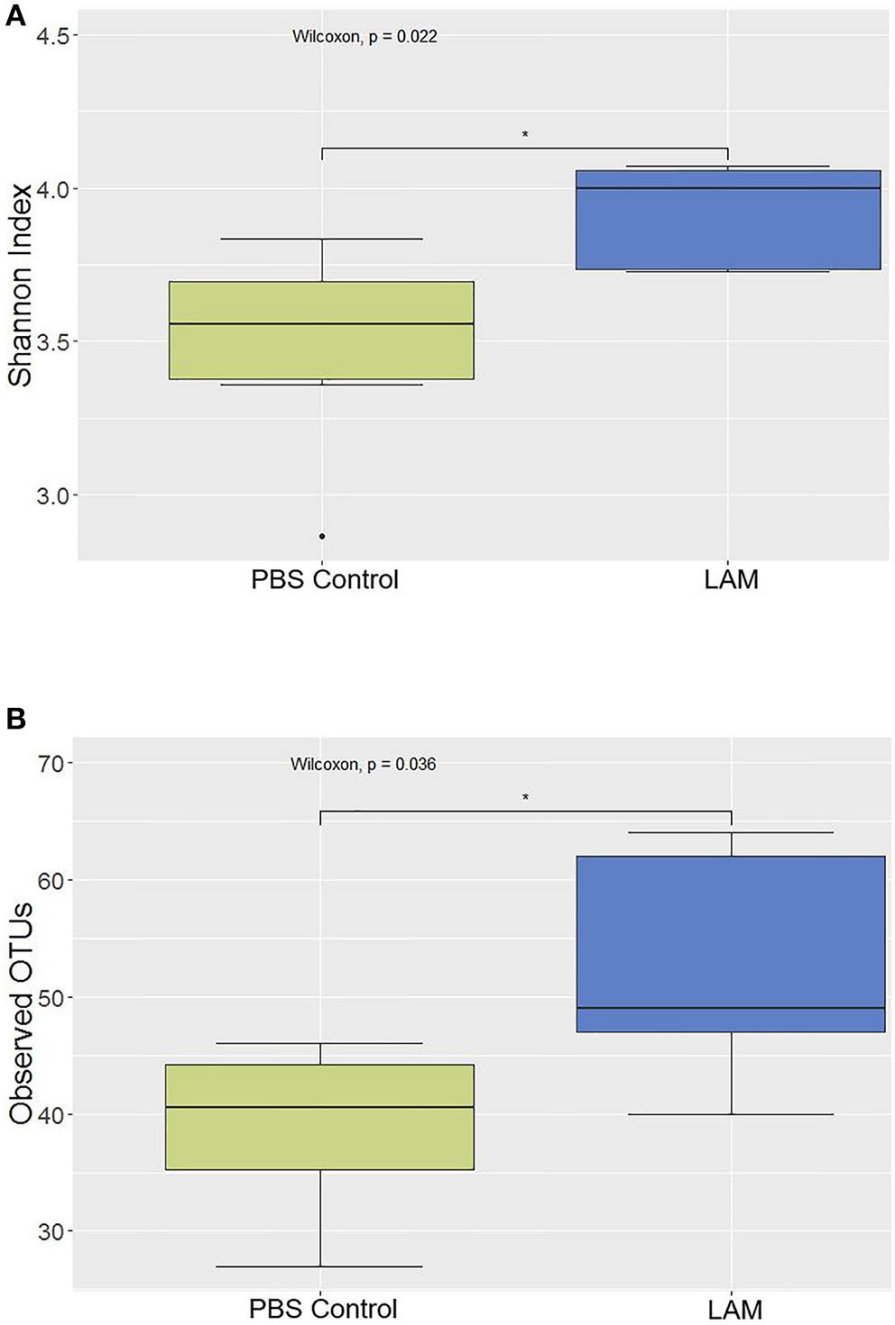
Alpha diversity between phosphate-buffered saline control and LAM as calculated by Shannon's diversity index **(A)** and observed OTUs **(B)** in trial 2. *In ovo* administration of LAM significantly increased the Shannon diversity **(A)** as well as species richness **(B)** when compared with those in the phosphate-buffered saline control *(Wilcoxon test, *P* < 0.05).

**Figure 7:**
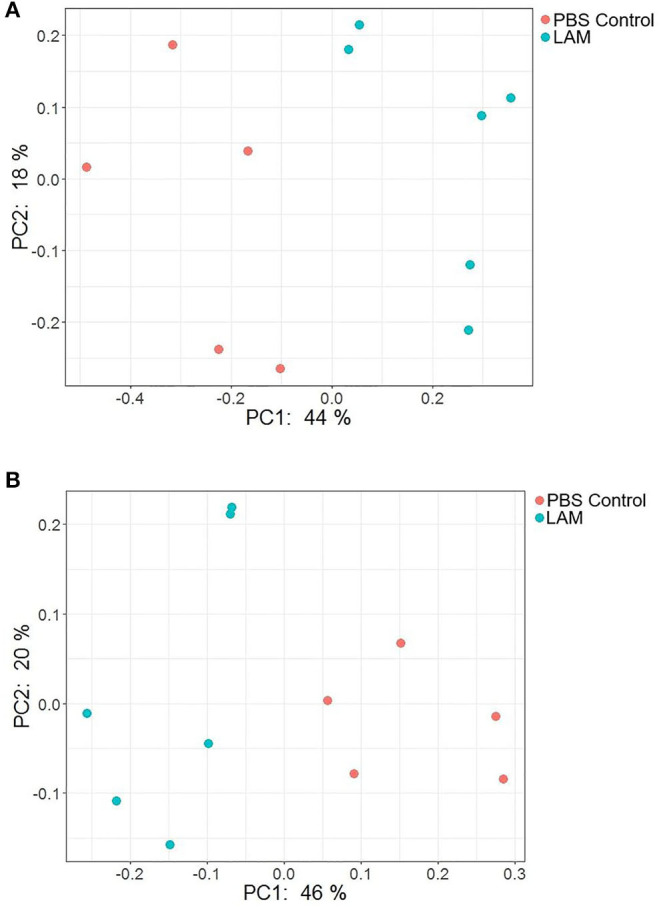
PCoA plot showing the bacterial community structure between phosphate-buffered saline control and LAM treatment groups as measured by the Bray–Curtis **(A)** and unweighted UniFrac **(B)** distance metric. There was a significant difference in the community structure between two treatment groups when measured with both metrics using permutational multivariate analysis of variance (Bray–Curtis, *P* = 0.003 and unweighted UniFrac distance metric; *P* < 0.001) in trial 2.

The alpha diversities, as measured by Shannon's diversity index and the observed OTU metric, are shown in Figures 6, 7, respectively. The alpha diversity calculated by both metrics was significantly higher in the LAM group as compared with that in the PBS control (Wilcoxon test, *P* < 0.05). This indicates that the species richness increases when treated with LAM as compared with PBS.

### Beta Diversity

The PCoA plots illustrating beta diversities as computed by Bray–Curtis and unweighted UniFrac distance metrics are shown in Figures 7A,B, respectively. In agreement with the alpha diversity, there was a significant difference in bacterial community structure between PBS control and LAM as measured by both Bray–Curtis (PERMANOVA, *P* = 0.003) and unweighted UniFrac (PERMANOVA, *P* < 0.001) distance metrics.

## Discussion

Several investigators have shown the significance of *in ovo* administration in the composition and diversity of the intestinal microbiota of neonate broiler chickens (4, 5, 29). Additionally, *Bacteroides, Clostridium* cluster XIVa, and *Clostridium* cluster IV have been described to have a profound role in intestinal homeostasis and reduction of inflammation (30, 31). In the present study, the bacterial composition at the family level revealed that *Enterobacteriaceae* was numerically higher in the PBS control group, whereas the *Ruminococcaceae* was significantly higher in the LAM treated group. *Ruminococcaceae* is a family in the class Clostridia, which includes *Clostridium* and other similar genera (Figures 2–5). *Ruminococci* spp. are among the most abundant cellulose-degrading bacteria in the rumen and may also make a significant contribution to plant cell wall breakdown in the large intestine in other mammals. They belong to the clostridial cluster IV, contributing to up to 20% of bacteria present in humans and are important short-chain fatty acid (SCFA) producers (32). SCFAs induce profound physiological responses in gut integrity and reduce inflammation (33, 34). Interestingly, *Ruminococcus* spp., *Faecalibacterium* spp., and *Lachnospiraceae* spp. are essential butyric acid contributors (35). Gastrointestinal inflammation has been associated with a significant reduction of *Clostridium* clusters XIVa and IV, such as *Lachnospiraceae, Ruminococcus*, and *Roseburia* (36), hence, the importance of differentiating beneficial clostridial strains from pathogenic strains such as *Clostridium perfringens* and *Clostridium difficile* (37). In the present study, SCFA-producing bacteria of the family *Ruminococcaceae* and the genus *Butyricicoccus* were not detected in the PBS control, whereas they were found in the LAM. Moreover, the unidentified genera that belong to *Lachnospiraceae* were significantly higher in embryos inoculated with LAM, whereas the *Enterobacteriaceae* family was significantly higher in embryos inoculated with PBS. *Lachnospiraceae* (phylum Firmicutes, class Clostridia) is abundant in the digestive tracts of many mammals and is crucial bacteria because of their role in the production of SCFA (38). In mice, probiotics have been shown to induce significant changes in SCFA. This, in turn, has a profound impact on intestinal physiology as well as pathogen control for enteropathogens such as enterohemorrhagic *E. coli* O157: H7 (39). In chickens, *in ovo* application of probiotics suggests that they can improve performance and immune functions and provide resistance against enteropathogens without affecting the hatchability of chickens (3, 14, 40, 41). Likewise, in the present study, hatchability was not affected by the *in ovo* treatment in both trials (Table 2). Moreover, embryos inoculated with LAM showed a significant reduction in the total number of gram-negative bacteria at DOH and d7. This reduction was also accompanied by a significant increase in total LAB at DOH in the LAM-treated group when compared with that in the PBS control (Table 2). These results were associated with significant differences in both beta diversity and alpha diversity, suggesting that the LAM treatment may drive large-scale changes in the microbial community structure and composition (Figures 6, 7) as has been published previously (4, 5, 29). In summary, *in ovo* administration of a defined LAM isolated from adult hens did not affect hatchability, improved BWG and reduced mortality at d7, induced variations in the cecae microbiota structure, and reduced *Enterobacteriaceae* colonization on a virulent *E. coli* horizontal infection model in broiler chickens.

## Data Availability Statement

The sequencing data of cecal microbiota is available on NCBI Sequence Read Archive (SRA) 309 under BioProject number PRJNA639394.

## Ethics Statement

The animal study was reviewed and approved by Institutional Animal Care and Use Committee (IACUC) at the University of Arkansas, Fayetteville. Protocol No. 17073.

## Author Contributions

Conceptualization: MA-N and GT-I. Formal analysis: GT and JL. Investigation: BG, BA, MA, CS, and GT. Methodology: CV, BS-C, and DH-P. Software: JL and GT. Supervision: GT-I and BH. Validation: DH-P and BS-C. Visualization: BH. Writing—original draft: MA-N and GT-I. Writing—review and editing: XH-V, CV, BH, and GT-I. All the authors reviewed, edited, and approved the manuscript.

## Conflict of Interest

Eco-Bio LLC employs MA-N, and uBiome, Inc., employs MA. The remaining authors declare that the research was conducted in the absence of any commercial or financial relationships that could be construed as a potential conflict of interest. The reviewer LB declared past co-authorship with several of the authors JL, GT, and BH to the handling Editor.
